# EV71 3C protease induces apoptosis by cleavage of hnRNP A1 to promote apaf-1 translation

**DOI:** 10.1371/journal.pone.0221048

**Published:** 2019-09-09

**Authors:** Mei-Ling Li, Jing-Yi Lin, Bo-Shiun Chen, Kuo-Feng Weng, Shin-Ru Shih, Jesse Davila Calderon, Blanton S. Tolbert, Gary Brewer

**Affiliations:** 1 Department of Biochemistry and Molecular Biology, Rutgers Robert Wood Johnson Medical School, Piscataway, NJ, United States of America; 2 Department of Clinical Laboratory Sciences and Medical Biotechnology, College of Medicine, National Taiwan University, Taipei, Taiwan; 3 Research Center for Emerging Viral Infections, Chang Gung University, Tao-Yuan, Taiwan; 4 Department of Medical Biotechnology and Laboratory Science, Chang Gung University, Tao-Yuan, Taiwan; 5 Department of Chemistry, Case Western Reserve University, Cleveland, OH, United States of America; University of British Columbia, CANADA

## Abstract

Enterovirus 71 (EV71) induces apoptosis to promote viral particle release. Earlier work showed that EV71 utilizes its 3C protease to induce apoptosis in a caspase-3-dependent pathway, though the mechanism is unknown. However, work from Vagner, Holcik and colleagues showed that host protein heterogeneous ribonucleoprotein A1 (hnRNP A1) binds the IRES of cellular apoptotic peptidase activating factor 1 (apaf-1) mRNA to repress its translation. In this work, we show that apaf-1 expression is essential for EV71-induced apoptosis. EV71 infection or ectopic expression of 3C protease cleaves hnRNP A1, which abolishes its binding to the apaf-1 IRES. This allows IRES-dependent synthesis of apaf-1, activation of caspase-3, and apoptosis. Thus, we reveal a novel mechanism that EV71 utilizes for virus release via a 3C protease–hnRNP A1–apaf-1–caspase-3–apoptosis axis.

## Introduction

Enterovirus 71 (EV71) is a positive-stranded RNA virus in the genus *Enterovirus*, family *Picornaviridae*. It is one of the major pathogens causing hand, foot and mouth disease (HFMD) particularly in the Asia-Pacific region. Some EV71 strains can lead to severe neurological complications ranging from aseptic meningitis with or without pulmonary edema to brain stem encephalitis and poliomyelitis-like acute flaccid paralysis, particularly among children under five years old [1–4]. Outbreaks of EV71 infection have occurred worldwide [5]. To date, there is no FDA-approved vaccine or antiviral agent against EV71.

Apoptosis is a highly regulated, programmed cell death to eliminate damaged, aged, or virally infected cells. EV71 infection can induce apoptosis in various cell types through different mechanisms [6]. For example, EV71 infection modulates the expression of miR-146a or miR-370 to induce apoptosis through targeting Son of sevenless homolog 1 (SOS1) and Growth arrest and DNA damage-inducible protein 45β (GADD45β) [7]. EV71 infection activates calpain via calcium flux to induce a caspase-independent apoptotic pathway [8]. EV71 2B protein (2B) induces cell apoptosis by recruiting the pro-apoptotic protein Bax to mitochondria and inducing Bax conformational activation [9]. Cleavage of eukaryotic initiation factor 4G (eIF4G) by EV71 2A protease also triggers apoptosis [10]. Some years ago, we also reported that EV71 3C protease triggers apoptosis through activation of caspase-3 [11]. However, the underlying molecular mechanisms involved in this event are not well understood.

Apoptotic peptidase activating factor 1 (apaf-1) plays an essential role in the apoptosis regulatory network. Upon binding cytochrome c and dATP, apaf-1 assembles into an oligomeric apoptosome which is responsible for autocatalytic activation of pro-caspase-9 (apaf-3). This in turn activates caspase-3 and apoptosis [12]. Our previous studies showed that EV71 infection activates caspase-9 and caspase-3 to induce apoptosis [6, 11]. The 5' UTR of apaf-1 contains an IRES for translation initiation to ensure continuous expression of apaf-1 despite an overall reduction of protein synthesis under apoptosis conditions [13–15].

Heterogeneous nuclear ribonucleoprotein (hnRNP) A1, an RNA-binding protein that shuttles between the nucleus and the cytoplasm, is involved in several RNA metabolic processes such as pre-mRNA splicing and RNA trafficking. hnRNP A1 is also an IRES trans-acting factor (ITAF) that binds to the IRES of EV71, human rhinovirus-2 (HRV-2), and apaf-1 mRNA to regulate their translation [16, 17]. Binding of hnRNP A1 to the IRES of EV71 and HRV-2 enhances translation, while binding to the apaf-1 IRES blocks its translation to inhibit apoptosis [16–18].

Essential to the viral life cycle, the 3C protease cleaves the EV71 polyprotein post-translation. In addition to processing viral polyprotein, 3C protease cleaves several host factors and impairs host transcription and translation. For example, 3C proteases of EV71, polio-, and human rhino viruses cleave the RNA-binding protein AUF1 to promote viral translation [19, 20].

Despite the observations described above, connections are lacking between EV71 infection, 3C protease, the ITAF hnRNP A1, IRES-dependent regulation of apaf-1 translation, and activation of apoptosis, which permits virus release. In the present study, we show that 3C protease cleaves hnRNP A1, thereby reducing hnRNP A1 association with the apaf-1 IRES. This allows apaf-1 translation and triggers apoptosis for virus release. Thus, our study reveals that, in addition to interacting with the EV71 IRES to promote viral translation, hnRNP A1 plays an important role in EV71 release through a novel mechanism the virus utilizes to induce apoptosis.

## Materials and methods

### Cell culture and EV71 infection

SF268 (human glioblastoma), RD (human embryonal rhabdomyosarcoma) and Vero (African green monkey kidney) cells were cultured as described [19]. EV71 (TW/2231/98) was propagated in RD cells. Cells were infected with EV71 at the indicated multiplicity of infection (moi) and then incubated at 37°C for 1 h for adsorption. Unbound virus was removed, and cells were refed fresh medium. Media from infected cultures were harvested at the indicated times, and virus titers were determined by plaque assay on Vero cells.

### Plasmid construction

Plasmid pRHF was constructed as described [18]. A bicistronic reporter plasmid, pRHF–apaf-1-5’ UTR, containing the apaf-1 5’UTR between the Renilla and Firefly luciferase coding regions, was constructed by inserting a Not I–apaf-1 5’UTR–Not I fragment into pRHF. In vitro transcription with this plasmid as template produces the bicistronic reporter RNA used for transfections.

### Western blot analysis

Western blotting was carried out as described [19]. Primary antibodies were used at the following dilutions: anti-hnRNP A1 mouse monoclonal [9H10] (Abcam), 1:1,000; anti-apaf-1 rabbit monoclonal [D5C3] (Cell Signaling Technology), 1:1,000; anti-eIF4G-I rabbit monoclonal [D6A6] (Cell Signaling Technology), 1:1,000; anti-3C protease mouse monoclonal [21], 1:50; anti-β actin rabbit polyclonal (Abcam), 1:15,000; anti-α-tubulin mouse monoclonal (Sigma-Aldrich), 1:500.

### Preparation and purification of recombinant protein

Recombinant wild-type and mutant 3C proteases were expressed in bacteria and purified as described previously [21].

### Cell transfections

For DNA transfection, cells were transfected using FuGENE 6 (Roche Diagnostics) following the supplier's instructions. Cells were subsequently incubated at 37°C for 24 h before harvesting and analysis. For RNA transfection, cells were transfected using Lipofectamine 2000 (Invitrogen, Carlsbad, CA) with 0.5 μg of bicistronic reporter RNA. Cells were incubated at 37°C for 2 days before harvesting and analysis.

### RNA interference (RNAi)

SF268 cells were seeded in 12-well plates in antibiotic-free medium. Transfections of small interfering RNA (siRNA) oligonucleotides against apaf-1 (ON-TARGETplus SMARTpool, catalog number L-003456-01-0005, Dharmacon), or control siRNA with scrambled sequence, were performed on 70–80% confluent cells with a siRNA concentration of 100 nM. siRNAs were transfected into SF268 cells with Oligofectamine (Thermo Fisher Scientific) according to the manufacturer's recommendations. Western blot analysis was performed 48 h post transfection.

### Electrophoretic mobility shift assay (EMSA)

For probe preparation, total RNA was extracted from SF268 cells and RT-PCR was carried out using primers specific to the apaf-1 5’UTR. The T7 promoter sequence was incorporated into the 5’ end of the upstream primer. In vitro transcription was carried out using the Maxiscript kit (Thermo Fisher Scientific) and [α-^32^P]UTP. Mixtures contained 1×10^4^ cpm of labeled RNA per reaction and were incubated at 37°C for 30 min either alone or with 1 μg of purified recombinant hnRNP A1. Reactions were fractionated in native polyacrylamide gels and RNA-protein complexes were visualized by phosphorimager.

### In vitro protease cleavage assay

Ten micrograms of 3C protease or an inactive C147S mutant was incubated with 30 μg cell lysate proteins in digestion buffer (20 mM HEPES, 20% glycerol, and 100 mM KCl, at pH  7.9) in a total volume of 20 μl at 37°C for four hours. Cleaved proteins were detected by Western blotting.

As a parallel method, hnRNP A1 was synthesized and labeled with [^35^S]-methionine using the TNT in vitro, coupled transcription/translation system (Promega). To examine cleavage of [^35^S]-labeled hnRNP A1, 4 μl of labeled hnRNP A1 reaction mixture was incubated with 5 μg of 3C protease in buffer (20 mM HEPES, 20% glycerol and 100 mM KCl at pH  =  7.9) with a total volume of 15 μl at 37°C for two hours.

### RNP-immunoprecipitation

Immunoprecipitations of endogenous protein-RNA complexes were used to assess association of hnRNP A1 with apaf-1 mRNA in cells as described [19]. For quantitations of mRNAs in precipitates, purified RNAs were reverse transcribed into cDNAs using the High Capacity cDNA Reverse Transcription kit (Applied Biosystems), followed by SYBR green quantitative PCRs. The forward and reverse primer sequences, respectively, are as follows: apaf-1: 5’-CAAGAAACGCCCGAAAGGGA-3’ and 5’-CCTGCTTTGGGCTCCCAC-3’; GAPDH: 5′-TTTAACTCTGGTAAAGTGGATATTGTTG-3′ and 5′-ATTTCCATTGATGACAA GCTTCC-3′.

### Cell death assay

For analysis of DNA fragmentation, DNA was isolated using the Blood and Cell Culture DNA minikit (Qiagen) and analyzed by agarose gel electrophoresis.

### Caspase assay

Caspase activity was analyzed using the ApoAlert Caspase Colorimetric Assay Kit (Clontech).

### Statistics

Data from three replicate experiments were compared using the unpaired two-tailed *t* test. *P* < 0.05 was considered significant. **P < 0.01; ***P < 0.001; N.S., not significant.

## Results

### EV71 infection decreases levels of hnRNP A1 via proteolytic cleavage by 3C protease

**Fig 1:**
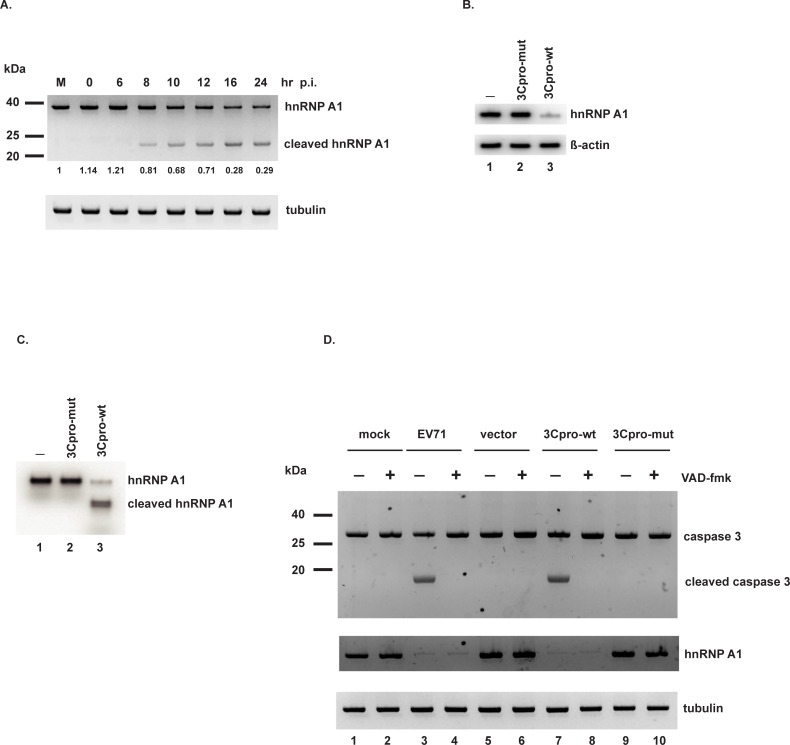
EV71 3Cpro cleaves hnRNP A1. (A) SF268 cells were infected with EV71 at an moi of 10 or mock infected for 24 hours (lane M). Cell lysates were collected at the indicated time points post-infection. Full-length and truncated hnRNP A1 levels were determined by Western blotting. Full-length hnRNP A1 was quantified and normalized to tubulin, the loading control. The level in mock-infected cells was set = 1.0. Numbers are listed under the blot for hnRNP A1 (B) Purified recombinant wild-type or mutant 3Cpro was added to cell lysates. Following incubation, hnRNP A1 abundance was determined by Western blotting. β-actin: loading control. (C) hnRNP A1 was labeled with [^35^S]-methionine by in vitro translation. In vitro cleavage assays were performed with wild-type or mutant 3Cpro. Proteins were resolved by 10% SDS-PAGE and detected by phosphorimager. (D) SF268 cells were mock infected or infected with EV71, or transfected with empty vector, or wild-type or mutant 3Cpro expression construct. Pan-caspase inhibitor zVAD-fmk (or DMSO vehicle) was added at a concentration of 50 μM to the indicated cultures for 1–2 days. Levels of caspase-3, cleaved caspase-3, and hnRNP A1 were determined by Western blotting. Tubulin: loading control.

In prior work, we established an in vitro cleavage assay using recombinant 3C protease (hereafter referred to as 3Cpro) and HeLa cell extract. Making use of 2-D electrophoresis and MALDI-TOF analysis, we identified eight nuclear proteins that are potential targets for 3Cpro cleavage. One of these is hnRNP A1 [21]. Picornavirus 3Cpro generally cleaves peptides at Gln/Gly junctions; analysis of the amino acid sequence of hnRNP A1 (http://www.cbs.dtu.dk/services/NetPicoRNA/) confirmed that numerous Gln/Gly junctions reside within hnRNP A1 and are thus potential 3Cpro cleavage sites. To test whether EV71 infection cleaves and affects the abundance of hnRNP A1, SF268 cells were infected with EV71 and degradation of hnRNP A1 at various time points was determined by Western blot. Quantitation of Fig 1A shows that hnRNP A1 levels were relatively constant at 6 hours and decreased ~20% by 8 hours post-infection, coincident with appearance of a truncated hnRNP A1 product. hnRNP A1 levels fell ~70% by 16–24 hours post-infection.

To verify cleavage of hnRNP A1 by EV71 3Cpro, we performed two in vitro cleavage assays using recombinant wild-type 3Cpro or the C147S mutant 3Cpro (which lacks proteolytic activity). In the first assay, purified recombinant proteases were incubated with SF268 cell lysate; control reactions contained no added proteases. Western blot analysis of reaction products showed that wild-type 3Cpro decreased the amount of full-length hnRNP A1 while neither the mutant 3Cpro nor control reaction had any effect (Fig 1B, compare lane 3 with lanes 1 and 2). β-actin levels were unaffected in all reactions, indicating specificity of proteolysis by wild-type 3Cpro. In the second in vitro cleavage assay, purified recombinant 3C proteases (or no added proteins as a control) were incubated with [^35^S]-methionine–labeled hnRNP A1. As shown in Fig 1C, wild-type 3Cpro substantially reduced full-length hnRNP A1 and generated a truncated product; mutant 3Cpro and the control reaction had no effects (compare lane 3 with lanes 1 and 2).

Our previous study showed that EV71 infection and expression of 3Cpro activate caspases to induce apoptosis [11]. To rule out the possibility of caspases as the protease that cleaves hnRNP A1, we infected cells with EV71 or transfected cells with 3Cpro expression constructs. Pan-caspase inhibitor zVAD-fmk or vehicle was added to the culture media to examine the effects on hnRNP A1 levels. As shown in Fig 1D, EV71 infection or wild-type 3Cpro expression activated caspase-3 (lanes 3 and 7). Addition of zVAD-fmk blocked the activation of caspase-3 (lanes 4 and 8). EV71 infection or 3Cpro expression reduced hnRNP A1 levels, and addition of zVAD-fmk did not block degradation of hnRNP A1 (compare lane 4 with lane 3, and lane 8 with lane 7). These results suggest that caspase activity is not responsible for cleavage of hnRNP A1. Taken together, the results of the in vitro cleavage assays and caspase inhibitor assays indicate that 3Cpro cleaves hnRNP A1 to reduce its levels during EV71 infection.

### Apaf-1 is required for EV71 to induce apoptosis

**Fig 2:**
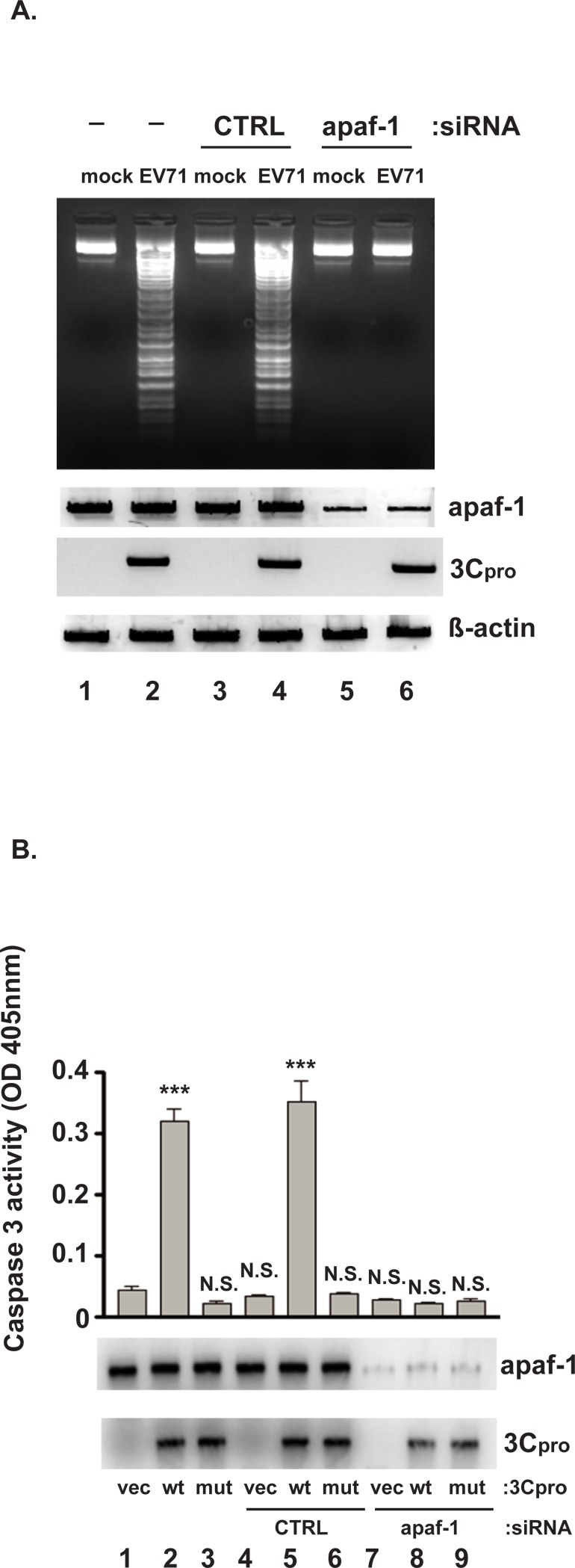
3Cpro-induced apoptosis requires apaf-1. (A) SF268 cells were transfected with either siRNAs targeting apaf-1 mRNA or control, scrambled siRNA (CTRL). Forty-eight hours post-transfection, cells were infected with EV71 at an moi of 10 or mock infected for 24 hrs. Extracted DNAs were fractionated by 1.5% agarose gel electrophoresis. Lower panels: Western blots of apaf-1 and 3Cpro. β-actin: loading control. (B) SF268 cells were transfected with either siRNAs targeting apaf-1 mRNA or control, scrambled siRNA. Forty-eight hours post-transfection, cells were transfected with plasmids expressing wild-type or mutant 3Cpro, or empty vector, for 2 days. Cell lysates were prepared for quantitative analysis of 3Cpro-induced caspase-3 activity. Mean values and standard errors from triplicate experiments are shown in the bar graphs. Levels of apaf-1 and 3Cpro were determined by Western blotting. ***P< 0.001; N.S., not significant.

EV71 infection triggers cell apoptosis to release viral particles. Apaf-1 interacts with cytochrome c to activate capsase-3, thus playing an essential role in apoptosis [22]. To determine if EV71 requires apaf-1 to induce apoptosis, the effects of apaf-1 knockdown on caspase-3 activity and DNA fragmentation in infected cells were examined. Endogenous apaf-1 levels were reduced by transfection of siRNAs targeting apaf-1 mRNA prior to infecting cells. Cells were then infected with EV71 and cellular DNA was extracted for agarose gel analysis. As shown in Fig 2A, DNA extracted from mock-infected SF268 cells was intact (lane 1), whereas DNA from EV71-infected cells presented the characteristic nucleosome ladder (lane 2). In cells transfected with control (CTRL), scrambled siRNA, no DNA fragmentation was observed in mock-infected cells (lane 3), while DNA fragmentation appeared in EV71-infected cells (lane 4), again as expected. By contrast, apaf-1 knockdown presented no DNA fragmentation in either mock- or EV71-infected cells (lanes 5 and 6, respectively). Western blotting confirmed apaf-1 knockdown compared to control siRNA and untransfected cells (compare lanes 5 and 6 with lanes 1–4), as well as 3Cpro expression in EV71-infected cells (lanes 2, 4, and 6). To assess the effect of apaf-1 knockdown on virus replication, virus titers were determined by plaque assay. Virus replicated normally in untransfected and control siRNA-transfected cells (1.2 x 10^8^ and 2 x 10^8^ pfu/ml respectively). Apaf-1 knockdown reduced virus titer 200- to 360-fold (to 5.6 x 10^5^ pfu/ml) compared to controls, indicating that blocking apoptosis severely inhibited virus release. These results indicate that apaf-1 is essential for EV71-induced apoptosis and effective virus release.

Additionally, our previous work showed that caspase-3 is involved in 3Cpro-induced apoptosis in infected cells [11]. This observation, combined with the requirement of apaf-1 for EV71-induced apoptosis (Fig 2A) prompted us to ask if apaf-1 links 3Cpro and caspase-3 to virus-induced apoptosis. If so, then depletion of apaf-1 should block activation of caspase-3 in cells expressing 3Cpro. To address this hypothesis, cells were first transfected with either control siRNA or siRNAs against apaf-1. Two days later, these cells were transfected with a plasmid expressing either wild-type 3Cpro or its C147S mutant. Caspase-3 activity was assayed 2 days later. Western blotting confirmed apaf-1 knockdown compared to control siRNA and untransfected cells (Fig 2B, compare lanes 7–9 with lanes 1–6), as well as 3Cpro expression in cells transfected with 3Cpro expression plasmids (lanes 2, 3, 5, 6, 8, and 9). As shown in Fig 2B, transfection of empty vector or plasmid expressing mutant 3Cpro did not induce caspase-3 activity in control siRNA-transfected cells (lanes 1, 3, 4, and 6), consistent with no apoptosis in the absence of 3Cpro (i.e., mock-infected cells in Fig 2A). While expression of wild-type 3Cpro induced caspase-3 activity in cells transfected with control siRNA (lane 5), apaf-1 knockdown abolished 3Cpro-induced caspase-3 activity (lane 8). This suggests that 3Cpro induces apoptosis through apaf-1–dependent activation of caspase-3 activity.

### A link between 3Cpro cleavage of hnRNP A1 and apaf-1 IRES activity

**Fig 3:**
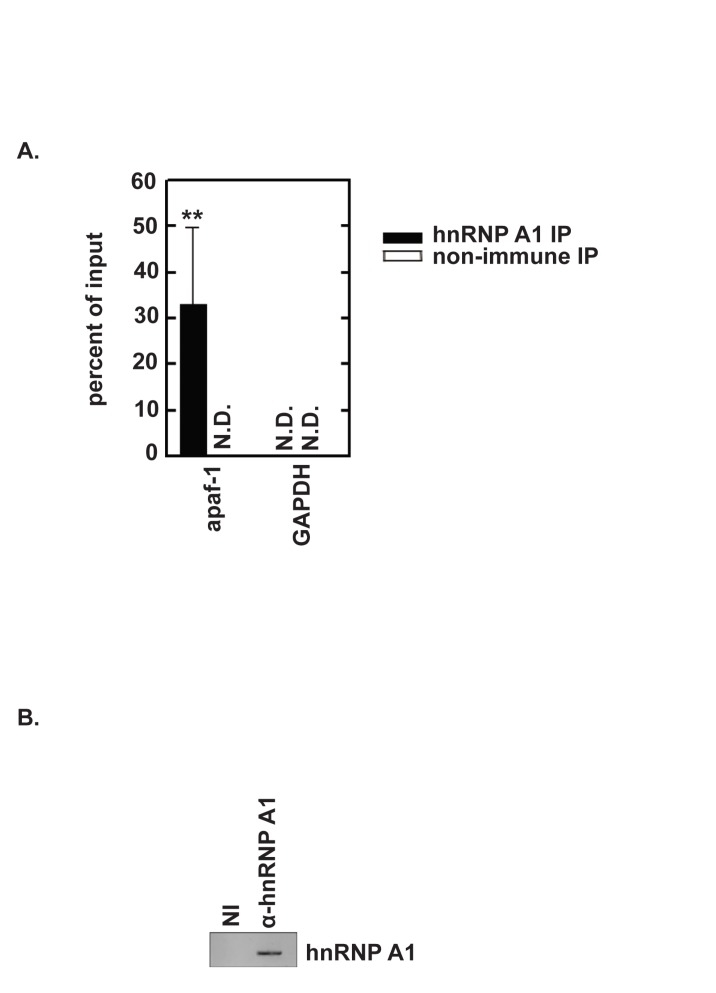
hnRNP A1 associates with apaf-1 mRNA in cells. (A) SF268 cell lysate was analyzed by ribonucleoprotein immunoprecipitation (RIP) with non-immune or anti-hnRNP A1 antibodies. Both input and immunoprecipitated materials were analyzed by qRT-PCR for apaf-1 mRNA and GAPDH mRNA (as a negative control). **P<0.01 for three independent RIP assays; N.D., not detected (B) Aliquots of immunoprecipitates were analyzed by Western blotting to confirm anti-hnRNP A1–dependent recovery of hnRNP A1.

Cammas et. al, [16] reported that hnRNP A1 relocates from the nucleus to the cytoplasm in response to UVC irradiation. In vitro experiments identified hnRNP A1 as an apaf-1 IRES-binding protein. Moreover, they showed that hnRNP A1 reduced UVC-triggered, apaf-1 IRES activity. In earlier work, we found that hnRNP A1 redistributes from the nucleus to the cytoplasm during EV71 infection [18]. We therefore speculated that in EV71-infected cells, cleavage of hnRNP A1 by 3Cpro reduces its binding to the apaf-1 IRES, thereby permitting translation of apaf-1 mRNA. Thus, hnRNP A1 cleavage relieves its inhibitory effect on apoptosis. To test this hypothesis, we first performed RNP immunoprecipitation and qRT-PCR to assess whether hnRNP A1 associates with apaf-1 mRNA in cells. SF268 cell lysates were prepared and RNA-protein complexes were immunoprecipitated using control, non-immune antibody or hnRNP A1 antibody. RNA isolated from immunoprecipitates was then analyzed by qRT-PCR. Apaf-1 mRNA was enriched by antibody against hnRNP A1 relative to non-immue (*P* < 0.01). GAPDH mRNA was not detected in immunoprecipitates from either antibody (Fig 3A). The Western blot in Fig 3B demonstrates that the hnRNP A1 antibody precipitated hnRNP A1 while the non-immune control did not precipitate any detectable hnRNP A1. These results suggest that hnRNP A1 associates with apaf-1 mRNA in cells.

**Fig 4:**
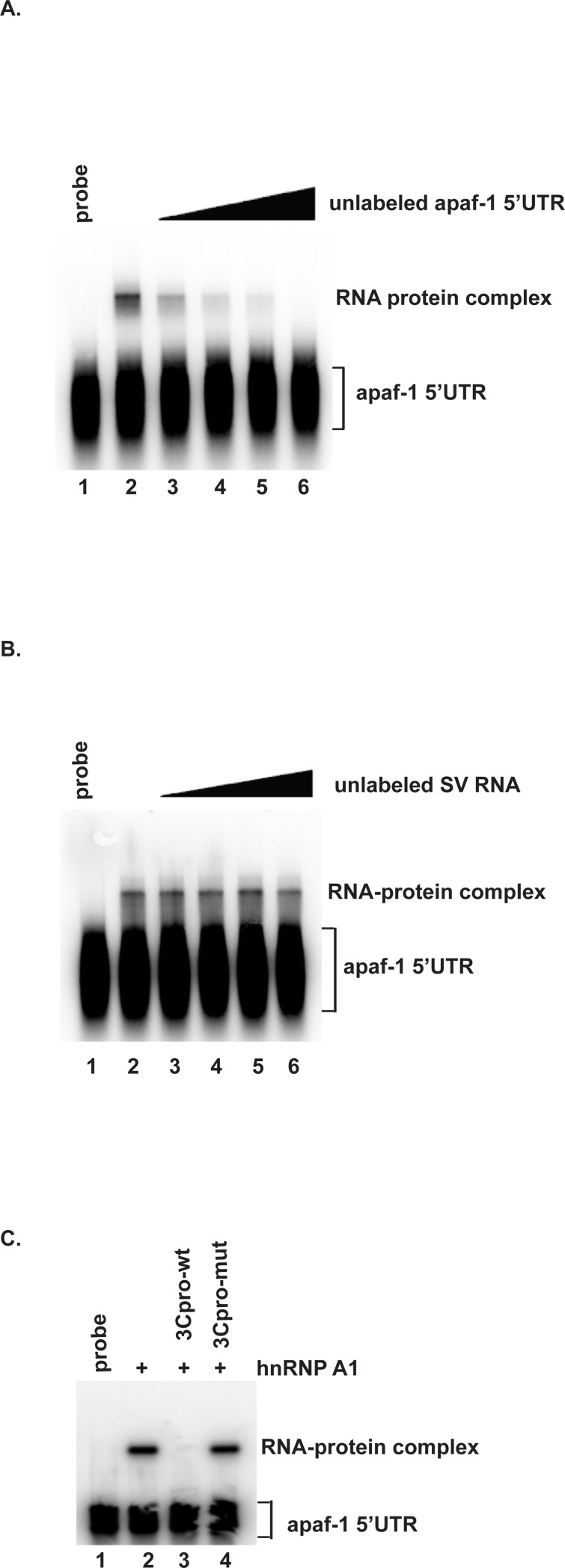
EV71 3Cpro inhibits binding of hnRNP A1 to the apaf-1 5’UTR. (A) Binding of hnRNP A1 to the apaf-1 5’UTR is specific. Radiolabeled apaf-1 5’UTR was incubated at 37°C for 30 min either alone (lane 1) or with 1 μg of purified recombinant hnRNP A1 (lanes 2–6). Lanes 3–6: 1x, 2x, 4x, and 8x molar excess of unlabeled apaf-1 5’UTR RNA were added to the reactions as a specific competitor. (B) 1x, 2x, 4x, and 8x molar excess of Sindbis virus RNA, which does not interact with hnRNP A1, were added to the binding reactions as a non-specific competitor RNA (lanes 3–6). (C) Effect of 3Cpro on hnRNP A1–apaf-1 5’UTR binding. EMSA was performed as described above following the addition of wild-type or mutant recombinant 3Cpro to RNA-protein binding reactions, as indicated.

To test whether hnRNP A1 directly associates with the apaf-1 5’UTR, EMSA was performed using purified recombinant hnRNP A1 and ^32^P-labeled apaf-1 5’UTR. As shown in Fig 4A, hnRNP A1 bound the apaf-1 5’UTR (lane 2). Addition of increasing amounts of unlabeled apaf-1 5’UTR decreased hnRNP A1 binding to the apaf-1 5’UTR (lanes 3–6). By contrast, addition of unlabeled Sindbis virus RNA, which does not interact with hnRNP A1, had no effect on hnRNP A1 binding to the apaf-1 5’UTR (Fig 4B). Thus, the competition assays indicate that the protein-RNA interaction is specific. Next, we examined the effect of 3Cpro on binding of hnRNP A1 to the apaf-1 5’UTR. EMSA was performed using purified recombinant hnRNP A1 and ^32^P labeled apaf-1 5’UTR, with the addition of recombinant wild-type 3Cpro or C147S mutant 3Cpro, or without addition of either 3Cpro. As shown in Fig 4C, hnRNP A1 bound the apaf-1 5’UTR and binding was abolished by addition of wild-type 3Cpro (compare lanes 2 and 3). Addition of C147S mutant 3Cpro had no effect on hnRNP A1 binding to the apaf-1 5’UTR (compare lanes 2 and 4). Together, these results indicate that hnRNP A1 interacts directly with the 5’UTR and hnRNP A1 cleavage by 3Cpro abolishes its 5’UTR-binding activity.

**Fig 5:**
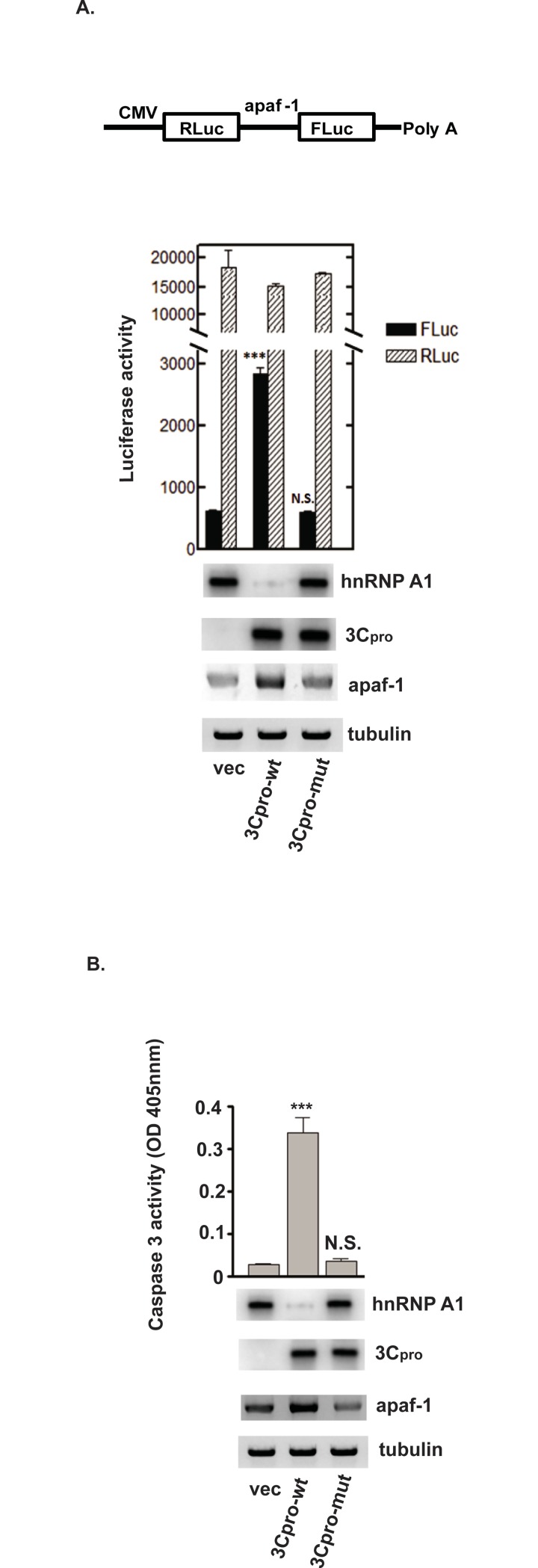
3Cpro cleavage of hnRNP A1 promotes apaf-1 IRES activity and apoptosis. (A) SF268 cells were transfected with plasmids expressing wild-type or mutant 3Cpro, or empty vector. Two days after transfection, RLuc–Apaf-1 5’UTR–FLuc bicistronic reporter RNA was transfected. Luciferase activity was measured 2 days after RNA transfection. Mean values and standard errors from triplicate experiments are shown in the bar graph. The diagram in the upper panel depicts the bicistronic reporter RNA. Western blotting was performed to examine the expression of 3Cpro, hnRNP A1, and apaf-1. Tubulin: loading control. (B) Caspase-3 activity was determined as described in the legend to Fig 2B following transfection of plasmids expressing either wild-type or mutant 3Cpro. Mean values and standard errors from triplicate experiments are shown in the bar graphs. ***P<0.001; N.S., not significant.

Cammas et al. [16] showed that UVC-induced nucleocytoplasmic redistribution of hnRNP A1 limited apaf-1 IRES activity; hnRNP A1 knockdown reversed this effect (i.e., knockdown increased IRES activity). As hnRNP A1 is a target of 3Cpro, we examined the effect of 3Cpro on apaf-1 IRES activity. Cells were transfected with a plasmid expressing wild-type or C147S mutant 3Cpro, or empty vector. Two days after transfection, RLuc–apaf-1 5’UTR–FLuc bicistronic reporter RNA was then transfected (Fig 5A, upper panel). Two days after reporter RNA transfections, IRES activity was determined by dual luciferase assay [23]. Levels of hnRNP A1, 3Cpro, and apaf-1 were determined by Western blotting. As shown in Fig 5A, expression of wild-type 3Cpro activated apaf-1 IRES activity ~fivefold (solid bar, lane 2, *P* < 0.001) and correlated with decreased hnRNP A1 level. As expected, cap-dependent RLuc activities were similar under all conditions (hatched bars, Fig 5A). Expression of the C147S mutant 3Cpro did not induce apaf-1 IRES activity or reduce abundance of hnRNP A1. Thus, expression of 3Cpro is sufficient to induce apaf-1 IRES activity. Western blotting was also performed to assess the consequence of hnRNP A1 cleavage by wild-type 3Cpro on cellular apaf-1 protein levels. Expression of wild-type 3Cpro increased apaf-1 protein levels ~twofold compared to mutant 3Cpro (compare lane 2 with lane 3) and correlated with increased apaf-1 IRES activity observed in the bicistronic reporter assay.

We next examined caspase-3 activation as a read-out to assess the consequence of hnRNP A1 cleavage by 3Cpro on induction of apoptosis. As shown in Fig 5B, expression of wild-type 3Cpro induced apoptosis as indicated by elevated caspase-3 activity; the mutant 3Cpro had no effect (compare lane 2 with lanes 3 and 1; *P*<0.001). Consistent with the results in Fig 5A, wild-type 3Cpro expression and hnRNP A1 destruction increased cellular apaf-1 expression ~twofold compared to mutant 3Cpro (compare lane 2 with lane 3). Taken together, our results indicate that, during EV71 infection, 3Cpro cleaves hnRNP A1, which increases apaf-1 IRES activity, and increases caspase-3 activity to trigger apoptosis.

**Fig 6:**
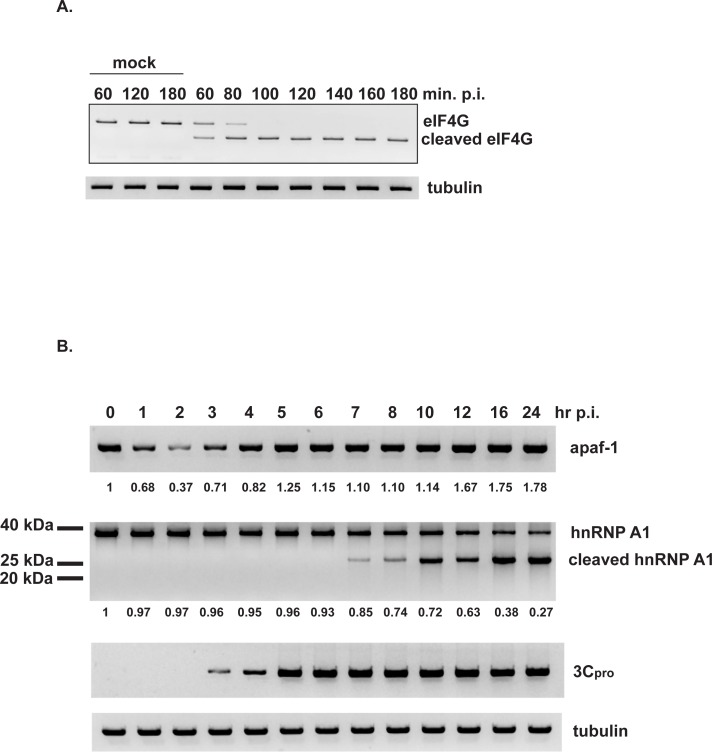
Time course of eIF4G cleavage and apaf-1 abundance following EV71 infection. SF268 cells were infected with EV71 or mock infected. At the indicated times post-infection, lysates were prepared for Western blotting for eIF4G (panel A); apaf-1, hnRNP A1, and EV71 3Cpro (panel B). Tubulin: loading control. Apaf-1 and full-length hnRNP A1 levels were normalized to tubulin for each time point with time-zero = 1. Numbers are listed below the respective blots for apaf-1 and full-length hnRNP A1.

As noted earlier, EV71 2A protease cleaves eIF4G [10]. This allows the virus to disrupt cap-dependent translation in infected cells and promote EV71 IRES-dependent translation and virus replication. As such, we assessed the levels and timing of expression of key proteins–eIF4G, 3Cpro, hnRNP A1, and apaf-1 –during a 24-hour time course of EV71 infection. Indeed, Western blotting of eIF4G in lysates of EV71-infected cells indicated that by 60 min. post-infection, ~50% of full-length eIF4G was cleaved; by 100 min, there was no detectable full-length eIF4G compared to mock-infected cells (Fig 6A). This would suggest that by 100 min., translation would shift from cap-dependent to cap-independent. Consistent with this conclusion, Fig 6B shows that the apaf-1 level decreased by ~60% (normalized to tubulin levels) by 2 hours post-infection. However, between 3–5 hours post-infection, apaf-1 levels increased and returned to near normal (i.e., the time-zero level). The cleavage profile of eIF4G is consistent with increasing apaf-1 protein levels beginning at 3 hours post-infection being due to apaf-1 IRES-dependent translation. 3Cpro was first detected at 3 hours post-infection, reached steady-state at 5 hours post-infection, and remained constant to 24 hours (Fig 6B). Thus, the earliest detectable time point for 3Cpro, at 3 hours, coincides with the time at which apaf-1 levels began to recover due to IRES-dependent translation. Apaf-1 levels remained relatively constant between 5–10 hours post-infection, and then increased ~70–80% above time-zero at late times post-infection, between 12 and 24 hours.

What is the effect of EV71 infection on hnRNP A1 expression versus time? Fig 6B shows that hnRNP levels were relatively constant between 1–6 hours post-infection and began to decline 7 hours post-infection, when the proteolytic cleavage product was first detected. It is during the late times post-infection, 16–24 hours, that hnRNP A1 fell to its lowest levels (Fig 6B), apaf-1 reached its highest levels (compared to time-zero; Fig 6B), and virus reached its highest titers (~8–9 x 10^8^ pfu/ml).

We conclude that hnRNP A1 cleavage does not appear to occur at early times (1–5 hours) when apaf-1 protein levels initially decline and then return to normal. However, EV71 utilizes hnRNP A1 and other ITAFs early in infection to promote EV71 IRES-dependent synthesis of virus proteins and virus replication [18]. Nonetheless, hnRNP A1 cleavage and further increases in apaf-1 levels occur at late times post-infection. That these late events during EV71 infection are important for apaf-1–induced apoptosis and virus release is demonstrated by the experiments showing that 3Cpro-dependent cleavage of hnRNP A1 induces apaf-1 IRES activity and increases levels of apaf-1 (Fig 5), a protein essential for apoptosis of infected cells (Fig 2). However, there may be additional mechanisms, albeit unknown ones, at early times post-infection that regulate cap-independent synthesis of apaf-1.

## Discussion

The EV71 3C protease plays an important role in viral protein maturation and virus-host interactions [24]. In addition to its RNA-binding activity [11], 3Cpro cleaves several host factors [21, 25–27]. We previously reported that 3Cpro activates caspase-3 to induce apoptosis in EV71-infected cells [11]. In the current study, we revealed the involvement of hnRNP A1 in EV71 3Cpro-induced apoptosis. We identified hnRNP A1 as a novel target of EV71 3Cpro. hnRNP A1 is one of the ITAFs that regulate IRES activity of some RNAs. Our current model is that hnRNP A1 binds to the apaf-1 IRES to block its translation [16]; in EV71-infected cells, 3Cpro cleaves hnRNP A1, thereby relieving its repressive effect to promote apaf-1 translation and apoptosis.

Early in infection, apoptosis serves as a host defense mechanism to restrict virus replication. Late in infection, viruses also induce apoptosis to release viral particles. Thus, it would benefit a virus to block apoptosis at early times after infection and allow it at later times. It has also been speculated that apoptosis resulting from tissue damage is related to EV71 pathogenesis. For example, Wang et al. reported that inhibition of apoptosis attenuated coxsackie virus B3 virulence [28]. Xi et al. demonstrated that inhibition of apoptosis decreased EV71 viral particle release [29]. Shi et al. utilized PCR arrays to examine expression of 84 apoptosis genes 8 and 20 hours after EV71 infection of human rhabdomyosarcoma (RD) cells. Apaf-1 gene expression declined fourfold by 8 hours and returned to normal at 20 hours compared to mock-infected cells [30]. We found that hnRNP A1 not only interacts with stem loops II and VI of the EV71 IRES to promote viral translation [17, 18, 23] but it also binds the apaf-1 IRES to inhibit its translation ([16] and the current work). Later in infection, 3Cpro cleaves hnRNP A1 resulting in apaf-1 translation and subsequent apoptosis for virus spreading. Our study and those of others suggest complex interactions between the host and EV71.

EV71 infection induces apoptosis through a variety of pathways. In addition to our findings, Li et al., [31] found that 3Cpro cleaves PinX1, a telomere binding protein, to promote apoptosis in EV71-infected cells. Song et al., [32] reported that the proteolytic activity of 3Cpro is required for the activation of caspase-9, caspase-8, and caspase-3. 3Cpro directly binds caspase-8 and caspase-9 but not caspase-3. A caspase-3 inhibitor prevents apoptosis in 3C-transfected cells. These findings imply the 3Cpro plays an important role in EV71-induced apoptosis.

In addition to proteolytic activity, 3Cpro has RNA-binding activity and binds to the first 126 nt of the EV71 5’UTR [11]. Some important questions to address in the future are, what are the consequences of the 3Cpro-EV71 5’UTR interaction? Does this interaction bring 3Cpro in close proximity to hnRNP A1 to cleave it? If so, does 3Cpro use a similar mechanism to bind the apaf-1 5’UTR and then cleave IRES-bound hnRNP A1? Finally, by what mechanism(s) does hnRNP A1 inhibit apaf-1 translation? Is apaf-1 mRNA sequestered in the nucleus with hnRNP A1 under non-infected conditions? Does relocalization of hnRNP A1 from the nucleus to the cytoplasm upon EV71 infection promote or inhibit interaction of hnRNP A1 and apaf-1 5’UTR?
